# Routine diagnostics for neural antibodies, clinical correlates, treatment and functional outcome

**DOI:** 10.1007/s00415-020-09814-3

**Published:** 2020-04-03

**Authors:** Christian G. Bien, Corinna I. Bien, Müjgan Dogan Onugoren, Desiree De Simoni, Verena Eigler, Carl-Albrecht Haensch, Martin Holtkamp, Fatme S. Ismail, Martin Kurthen, Nico Melzer, Kristina Mayer, Felix von Podewils, Helmut Rauschka, Andrea O. Rossetti, Wolf-Rüdiger Schäbitz, Olga Simova, Karsten Witt, Romana Höftberger, Theodor W. May

**Affiliations:** 1 Epilepsy Center Bethel, Krankenhaus Mara, Maraweg 17-21, 33617 Bielefeld, Germany; 2 Laboratory Krone, Bad Salzuflen, Germany; 3 Department of Neurology, Epilepsy Center, Friedrich-Alexander-Universität, Erlangen-Nürnberg, Germany; 4 Division of Neuropathology and Neurochemistry, Department of Neurology, Medical University of Vienna, Vienna, Austria; 5 Department of Neurology, University Hospital St. Poelten, St. Poelten, Austria; 6 Department of Neurology, Städtisches Klinikum Ludwigshafen Am Rhein, Ludwigshafen, Germany; 7 Department of Neurology, Kliniken Maria Hilf Moenchengladbach, Faculty of Health, University of Witten/Herdecke, Moenchengladbach, Germany; 8 Epilepsy-Center Berlin-Brandenburg, Institute for Diagnostics of Epilepsy, Evangelisches Krankenhaus Königin Elisabeth Herzberge, Berlin, Germany; 9 Department of Neurology, University Hospital Bochum, Knappschaftskrankenhaus, Bochum, Germany; 10 Swiss Epilepsy Center, Zurich, Switzerland; 11 Department of Neurology with Institute of Translational Neurology, University Hospital Münster, Münster, Germany; 12 Department of Neurology, University Hospital of Augsburg, Augsburg, Germany; 13 Department of Neurology, University Medicine Greifswald, Greifswald, Germany; 14 Department of Neurology and Karl Landsteiner Institute for Neuroimmunological and Neurodegenerative Disorders, Sozialmedizinisches Zentrum Ost, Donauspital, Vienna, Austria; 15 Department of Clinical Neurosciences, University Hospital (CHUV) and University of Lausanne, Lausanne, Switzerland; 16 Department of Neurology, EvKB-Bethel, Bielefeld, Germany; 17 Protestant Hospital Alsterdorf, Epilepsy Center Hamburg, Hamburg, Germany; 18 Department of Neurology and Research Centre of Neurosensory Sciences, Carl Von Ossietzky University, Oldenburg, Germany; 19 Society of Epilepsy Research, Bielefeld, Germany

**Keywords:** Neural autoantibodies, Autoimmune encephalitis, Laboratory test evaluation, Immunotherapy, Outcome

## Abstract

**Objective:**

To determine frequencies, interlaboratory reproducibility, clinical ratings, and prognostic implications of neural antibodies in a routine laboratory setting in patients with suspected neuropsychiatric autoimmune conditions.

**Methods:**

Earliest available samples from 10,919 patients were tested for a broad panel of neural antibodies. Sera that reacted with leucine-rich glioma-inactivated protein 1 (LGI1), contactin-associated protein-2 (CASPR2), or the voltage-gated potassium channel (VGKC) complex were retested for LGI1 and CASPR2 antibodies by another laboratory. Physicians in charge of patients with positive antibody results retrospectively reported on clinical, treatment, and outcome parameters.

**Results:**

Positive results were obtained for 576 patients (5.3%). Median disease duration was 6 months (interquartile range 0.6–46 months). In most patients, antibodies were detected both in CSF and serum. However, in 16 (28%) patients with *N*-methyl-d-aspartate receptor (NMDAR) antibodies, this diagnosis could be made only in cerebrospinal fluid (CSF). The two laboratories agreed largely on LGI1 and CASPR2 antibody diagnoses (*κ* = 0.95). The clinicians (413 responses, 71.7%) rated two-thirds of the antibody-positive patients as autoimmune. Antibodies against the α-amino-3-hydroxy-5-methyl-4-isoxazolepropionic acid receptor (AMPAR), NMDAR (CSF or high serum titer), γ-aminobutyric acid-B receptor (GABABR), and LGI1 had ≥ 90% positive ratings, whereas antibodies against the glycine receptor, VGKC complex, or otherwise unspecified neuropil had ≤ 40% positive ratings. Of the patients with surface antibodies, 64% improved after ≥ 3 months, mostly with ≥ 1 immunotherapy intervention.

**Conclusions:**

This novel approach starting from routine diagnostics in a dedicated laboratory provides reliable and useful results with therapeutic implications. Counseling should consider clinical presentation, demographic features, and antibody titers of the individual patient.

**Electronic supplementary material:**

The online version of this article (10.1007/s00415-020-09814-3) contains supplementary material, which is available to authorized users.

## Introduction

Neural immunoglobulin G (IgG) autoantibodies help to define or refine the diagnosis of autoimmune diseases of the central and peripheral nervous system (CNS, PNS) [19, 20, 24, 35]. These antibodies provide an understanding of the pathogenesis of these conditions [10], suggest adequate therapies, and permit prognostic estimates [12]. Most of our knowledge comes from reports by research laboratories on patients with specific antibodies. Much autoantibody testing in the world is done by diagnostic laboratories that investigate material from poorly selected patients, must rely on what is sent in (serum, cerebral spinal fluid [CSF], or CSF–serum pairs), utilize ready-to-use assays, and are expected to provide test results within a short timeframe. The added value of data from such a laboratory that it is closely connected to its clinical senders may be a “real-world” impression of frequencies, clinical correlates, and therapeutic and prognostic implications of neural antibodies. These factors may also further clarify the relevance of *N*-methyl d-aspartate receptor (NMDAR) antibodies in serum only. Here, we retrospectively evaluated the 4-year experience of one such diagnostic laboratory to address these issues.

## Methods

### Antibody testing

Sera, CSF samples, or CSF–serum pairs (latency CSF–serum collections: ≤ 7 days) received by the antibody laboratory in Bethel between 12/2011 and 12/2015 were analyzed. They had been transferred by clinicians for suspected neuropsychiatric autoimmune conditions or for confirmation of external antibody findings. Only the first material of a given patient sent to this laboratory was included. Some patients have been reported before [5–7, 14–16, 39].

#### Antibodies against surface antigens or glutamic acid decarboxylase 65 kDa (GAD65)

Antibodies were detected using commercially available biochips (Euroimmun, Lübeck, Germany). These cell-based assays (CBA) consist of human embryonic kidney (HEK-293) cells transfected with plasmids that encode the following antigens. Cells that express NMDAR with NR1 subunits only and GAD65 were fixed with acetone; cells that express leucine-rich glioma-inactivated protein 1 (LGI1), contactin-associated protein-2 (CASPR2), α-amino-3-hydroxy-5-methyl-4-isoxazolepropionic acid receptor subunits 1 and 2 (AMPAR1 and AMPAR2), γ-aminobutyric acid-B receptors (GABABR), or glycine receptor (GlyR) were fixed with paraformaldehyde. Their preparation follows an established principle [11] and has been described [46]. The protocol for indirect immunofluorescence followed the manufacturer’s recommendations (Euroimmun, FA 112d-1005-1, IgG) with these modifications (CGB, detailed in [4]): serum diluted 1:16; buffer was phosphate-buffered saline (PBS); secondary antibody was goat-anti-human IgG heavy and light chain (H + L) conjugated with Alexa Fluor 594 (Jackson ImmunoResearch, West Grove, PA, USA, No. 109-585-088), used at a dilution of 1:100 and incubated for 30 min at room temperature (RT); nuclear counterstaining with Hoechst 33,342, 1:10,000; embedding with 1,4-Diazabicyclo[2.2.2]octan. Stained biochips were examined using a fluorescence microscope (Leica DM 2000, Wetzlar, Germany) equipped with adequate filters. One of three neurologists experienced in the reading of this assay (CGB, CIB, or MDO) decided whether an antibody was present using the signal of the surrounding (supposedly negative) fields as respective negative controls. IgG positivity was confirmed by goat-anti-human antibody against the Fcγ fragment of IgG (conjugated with Alexa Fluor 488; Jackson Immunoresearch, No. 304-585-008) diluted 1:100 and incubated for 30 min at RT. Positive samples were endpoint titrated with the Fc antibody. These titrations were 1:16, 1:32, 1:64, and so on for serum and 1:1 (undiluted), 1:2, 1:4 and so on for CSF. Two of four investigators (CGB, CIB, MDO, or an experienced technician) determined the dilution that provided the last specific signal. This was noted as the titer. In cases of divergent ratings, the mean of the two ratings was recorded. We determined IgG subclasses as described before [6].

All samples were also tested on unfixed sagittal mouse brain slices that contained hippocampus, brain stem, and cerebellum (Euroimmun, Lübeck, Germany). These tissue-based assays (TBAs) were incubated with serum diluted 1:40 or undiluted CSF. More intense binding to the neuropil compared to cell bodies in hippocampus or cerebellum was noted as “neuropil staining”. Such reactivity was not required to make the diagnosis of a specified surface antibody; specific binding to transfected cells was sufficient.

### Categorization and analyses

Patients were categorized by their antibody results (Table 1). Data on NMDAR-high, LGI1, CASPR2, and GAD65 patients are preferentially described. We considered them as “major antibody groups”, given their frequency in the literature [12] and in this study. For rarer antibodies, see the Supplementary material.

**Table 1:** Antibodies: categorization for the purposes of this study, and frequencies

Category	Description	A	B	C	D	E	F	G
		*N*	% of ab-positive patients	% of total patients	Clinical rating available	Clinical rating in ab positives available (D/A)	Positive clinical rating	Serologically and clinically positive (F/D)
GAD65	GAD65 abs > 1:500 in serum or positive in CSF	119	20.7	1.09	93	78%	51	55%
VGKC complex	VGKC complex abs > 100 pM, not directed against LGI1 or CASPR2	90	15.6	0.82	66	73%	21	32%
LGI1	LGI1 abs at any titer in CSF or serum	81	14.1	0.74	49	60%	44	90%
Neuropil–CSF	Neuropil staining by CSF (regardless how serum behaved) and no specific surface ab found, with or without VGKC complex abs	74	12.8	0.68	49	66%	13	27%
NMDAR-high	NMDAR abs > 1:500 in serum or CSF positive, with or without VGKC complex abs	67	11.6	0.61	44	66%	42	95%
CASPR2	CASPR2 abs ≥ 1:128 in serum or CSF positive	46	8.0	0.42	39	85%	27	69%
Onconeural	Hu, Ma2, Yo, amphiphysin, CV2, Sox1 alone or in combination, in serum or in CSF	41	7.1	0.38	29	71%	17	59%
GABABR	GABABR abs at any titer in CSF or serum, with or without an accompanying onconeural ab	16	2.8	0.15	12	75%	11	92%
NMDAR with a low titer in serum and no CSF tested	NMDAR abs in serum ≤ 1:500 and no CSF tested	15	2.6	0.14	13	87%	5	38%
GlyR	GlyR abs at any titer in CSF or serum	13	2.3	0.12	10	77%	4	40%
NMDAR in serum only (negative in CSF)	NMDAR abs in serum but not in CSF	7	1.2	0.06	7	100%	4	57%
AMPAR2	AMPAR2 abs at any titer in CSF or serum	4	0.7	0.04	2	50%	2	100%
LGI1 + CASPR2	LGI1 abs at any titer in CSF or serum + CASPR2 abs ≥ 1:128 in serum or CSF positive	2	0.3	0.02	0	0%	–	–
NMDAR-high + GAD65	NMDAR-high + GAD65 abs > 1:500 in serum or positive in CSF	1	0.2	0.01	1	100%	0	0%
AMPAR1	AMPAR1 abs at any titer in CSF or serum	0	0.0	0.00	0	–	–	–
Positive	All ab positives	576	100.0	5.28	413	72%	241	58%

The remaining 10,343 were considered “negative”. This comprised: No ab; CASPR2 abs < 1:128 in serum and negative in CSF; GAD65 abs ≤ 1:500 in serum and negative in CSF; neuropil binding with serum only (CSF without neuropil abs or not investigated); onconeural abs positive by only one technique (tissue-based assay or immunoblot)

*Ab/abs* antibody/antibodies

### Interlaboratory reproducibility

All available sera with antibodies against LGI1, CASPR2, or the VGKC complex (as indicator of potential LGI1 or CASPR2 reactivity) plus 75 neighboring samples that did not harbor any of these antibodies (negative controls) were tested for LGI1 or CASPR2 antibodies by RH, who only knew that the samples were positive for at least one of the respective antibodies. In the first step, sera were screened with an in-house TBA. For this process, fresh adult rat brains were fixed in 4% paraformaldehyde for 1 h at 4 °C, cryoprotected with 40% sucrose for 48 h, embedded in freezing medium, and snap frozen in isopentane chilled with liquid nitrogen. Sagittal cryostat sections (7 μm) were defrosted for 15–30 min, washed in PBS, incubated for 15 min in 0.3% H_2_O_2_, and blocked for 60 min with 5% donkey serum in PBS. After incubation with patient’s serum (dilution: 1:200) at 4 °C overnight and labeling with secondary antibody (biotinylated donkey anti-human, 1:2000, 1 h, RT), slices were incubated with avidin–biotin for 1 h, visualized with 3.3-diaminobenzidin-tetrachlorid (DAB) for 7 min, and covered with coverslips. Samples with positive neuropil staining in the hippocampus and molecular layer of the cerebellum were further tested on a commercially available biochip (Euroimmun), according to the manufacturer´s instructions. A positive result in both TBA and CBA was required for the diagnosis of a surface antibody.

### Delivery notes

The following pieces of information from the delivery notes were documented: demographic data, tentative diagnoses (free text), and date of disease onset. From 12/2014 to 12/2015, senders were also asked to categorize the patients according to this list: encephalitis, epilepsy, cognitive/psychiatric problem, other disorder; multiple selections were possible.

### Questionnaires

The institutions who received positive antibody results were asked to complete a questionnaire with the following questions: date of questionnaire completion, previous viral encephalitis, antibody diagnosis known when material sent to this laboratory, date of disease onset, final diagnosis (free text), final judgement (CNS or PNS autoimmune disease), tumor, modified Rankin Scale (mRS) at antibody diagnosis and most recent follow-up, date of most recent follow-up. As follow-up, only visits ≥ 3 months from antibody diagnosis were accepted.

### Clinical syndromes

Clinical syndromes were defined post hoc by CGB based on free text notes on the questionnaires and the delivery forms.

### Ethics statement

The study was approved by the Ethics committee of the University of Münster, Germany (2017-005-f-S).

### Statistics

Kappa coefficient and Mann–Whitney *U* test were used as indicated. Statistical analyses were performed with IBM SPSS (Version 25).

### Data availability

Any data not published within this article are available at the Epilepsy Center Bethel. Data will be shared upon request from any qualified investigator, maintaining anonymization of the patients.

## Results

### Antibody-positive patients

Of the total 10,919 patients, 5592 were investigated in CSF–serum pairs (51.2%), 4803 for serum only (44.0%), and 524 for CSF only (4.8%). Mean age was 47.3 years (standard deviation [SD] 22.5, range 0.1–95 years; < 18 years: *N* = 1424 [13.0%]; females/males/sex unknown: 5696/5205/18, i.e., 52.3%/47.7%/0.2%); > 90% of results were returned within one week. For 3434 patients, 3481 clinical categorizations were noted: encephalitis (38.1%); epilepsy (18.6%); cognitive/psychiatric problem (1.0%); other (31.9%); no information (11.7%); multiple answers were possible. VGKC complex antibodies were assessed in 9239 cases (84.6%).

**Fig. 1:**
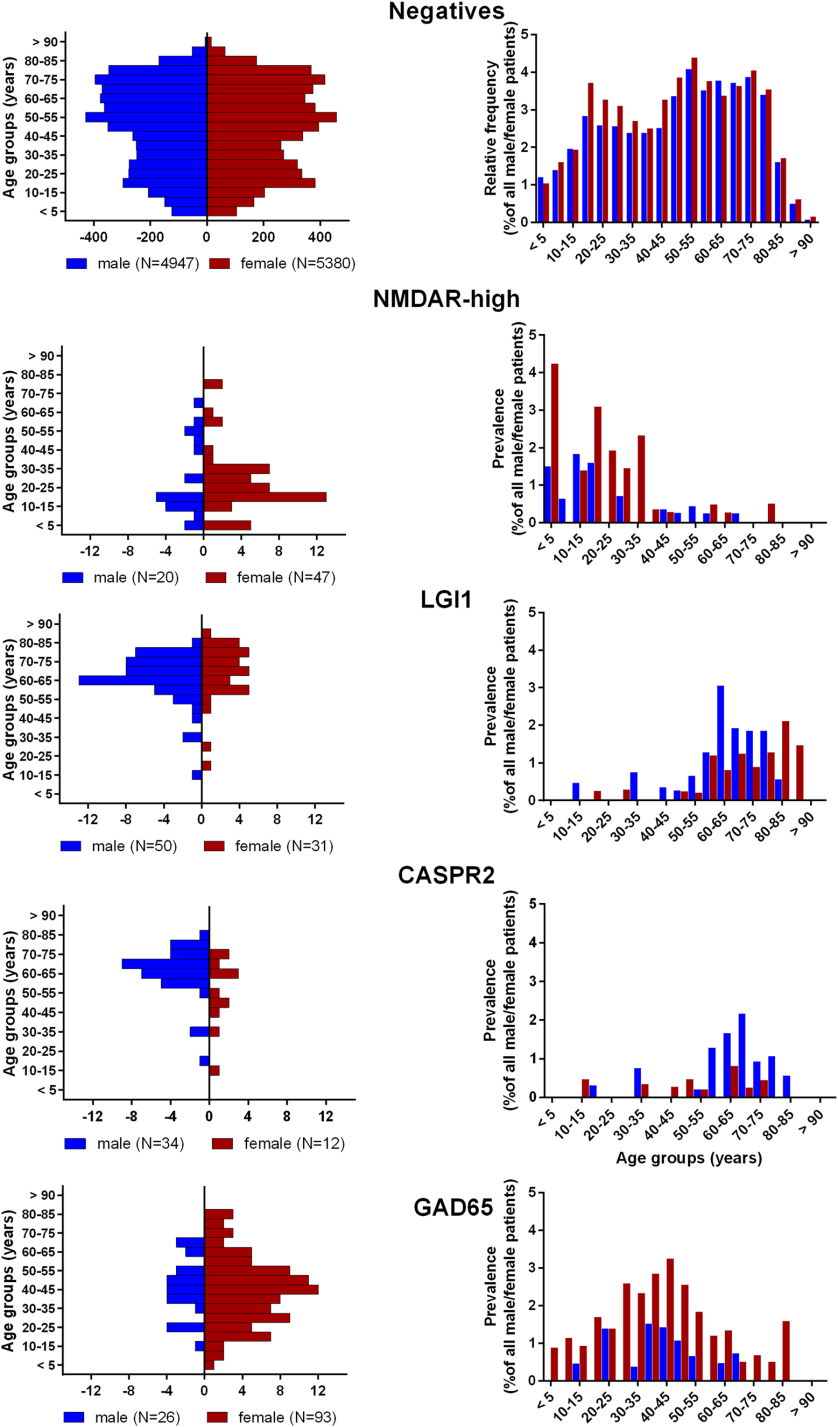
Age, sex, and prevalence. Distribution of age (in 5-year-intervals) and sex (red = female, blue = male) in the four major groups and the negatives. In the diagrams to the right, the bars indicate antibody prevalences, i.e., the proportions of positives related to all investigated patients (divided into subgroups defined by age and sex)

Neural antibodies were diagnosed in 576 patients (5.3%; Table 1). In 59 patients (10.2%), these results confirmed previous external antibody findings. The female/male/sex unknown percentages among the antibody-positive cases were 54.8%/45.0%/0.2%. For age and sex distributions, see Fig. 1.

**Fig. 2:**
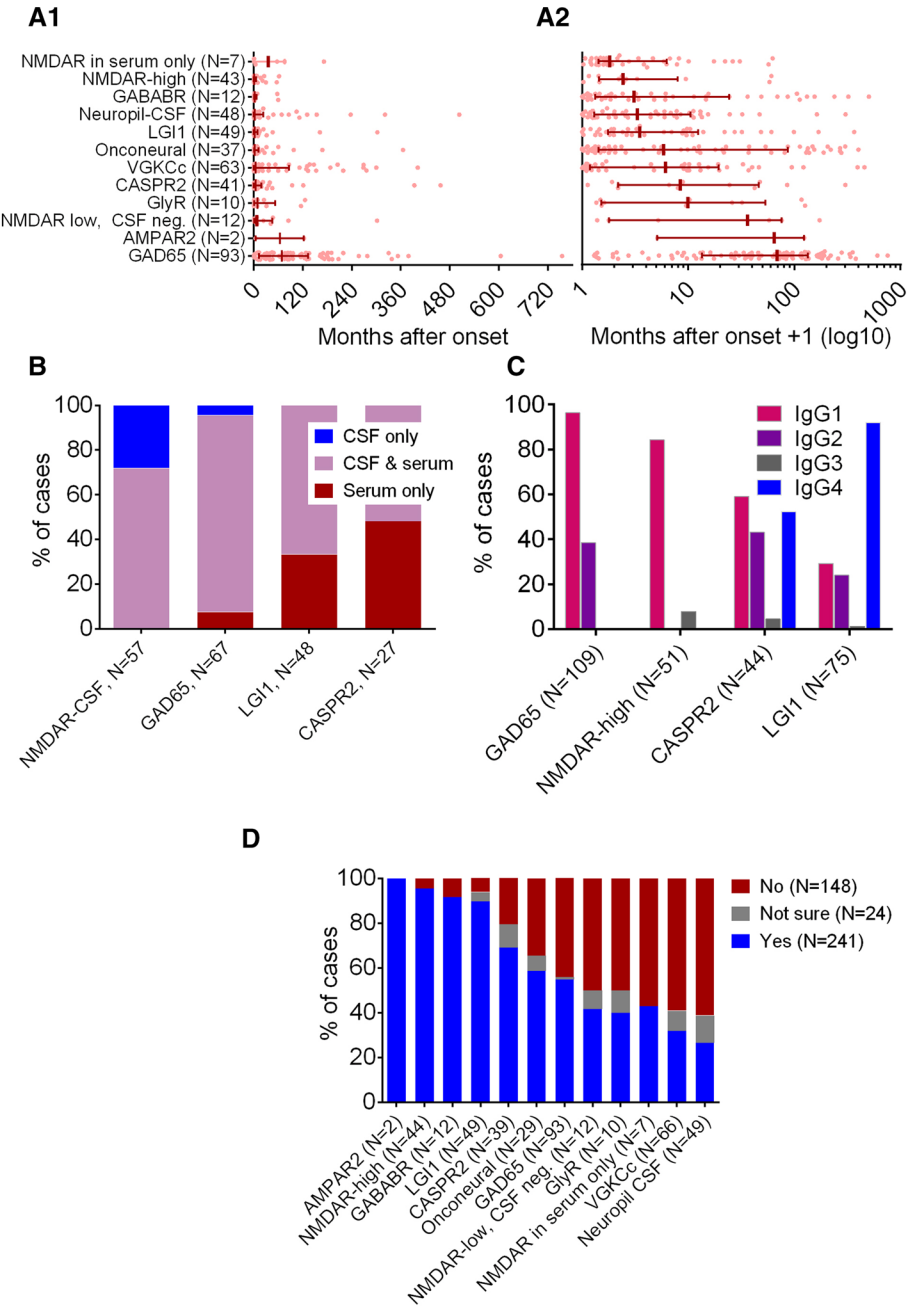
Disease durations, CSF/serum/CSF–serum pairs, IgG subclasses, and clinical ratings. **a** Latency (in months) between disease manifestation and antibody diagnostics in the antibody-positive cases with known disease onset. The lines indicate medians with quartiles. Antibody groups are given in ascending order of their median latencies. *A*_1_: linear *x*-axis, *A*_2_: logarithmic *x*-axis (note that “1” was added to all values to be able to include values of zero). **b** Ratio of cases with serum-only or CSF-only antibody positivity in the four major antibody groups plus onconeural and GABABR antibodies. The small groups with serum-only and CSF-only findings in the GAD65 group are cases with either very low CSF titers (*N* = 3) and negative serum or serum titers of just 1:500 and negative CSF samples (*N* = 2). In the onconeural group, there was one Ma2 case that was not fully appreciated in serum (blot positivity only) but clearly diagnosed in CSF (blot and tissue-based assay positive). **c** IgG subclasses in the four major antibody groups. **d** Clinical retrospective ratings (“Autoimmune disease of the CNS or PNS?”) in descending order of the positive ratings

In the patients with this piece of information available (*N* = 417), materials were sent after a median disease duration of 6 months (interquartile range [IQR] 0.6–46, mean 46, SD 92 months, maximum 63 years); for the single antibody groups, see Fig. 2a. The NMDAR-high group stood out, because in 16 (28%) cases, antibodies were detected in CSF only. In contrast, LGI1 and CASPR2 patients were identified by serum-only positivity in 33% and 48% of cases, respectively (Fig. 2b). The proportions of the subclasses for the four major groups (*N* = 279) are shown in Fig. 2c.

### Interlaboratory reproducibility of antibody test results

There was an agreement of 97.3% (223/229) with *κ* = 0.952 (*p* < 0.001; Table 2).

**Table 2:** Comparison of test results from Bethel and Vienna in 229 patients with antibodies against elements of the VGKC complex

	Vienna				Total
	LGI1/CASPR2 negative	LGI1	CASPR2	LGI1 + CASPR2	
Bethel					
LGI1/CASPR2 negative	136*	0	1^1^	0	137
LGI1	1^2^	67	0	0	69
CASPR2	2^3, 4^	0	19	2^5, 6^	23
LGI1 + CASPR2	0	0	0	1	0
Total	139	67	20	3	229

Grey cells: matches between labs; cells to the left of grey cells: Bethel “more sensitive” or “less specific”; cells to the right of grey cells: Vienna “more sensitive” or “less specific”. *κ* = 0.952; *p* < 0.001

*Seventy-five of these 136 results had VGKC complex antibodies < 100 pM (i.e., in the normal range). They were included as negative controls. None of them were found to be LGI1- or CASPR2-antibody-positive by either laboratory

^1^75 years, female, Bethel: high-titer GABABR and Sox1. VGKC complex 100 pM. Paraneoplastic limbic encephalitis, outcome: mRS unchanged

^2^64 years, male, Bethel: LGI1 1:64, VGKC complex < 1 pM. Faciobrachial dystonic seizures and limbic encephalitis, mRS -4 (improved)

^3^60 years, female, Bethel: CASPR2 1:250 in serum, CSF negative, VGKC complex 155 pM. No clinical data

^4^75 years, female, Bethel: CASPR2 1:250 in serum, CSF not studied, VGKC complex 217 pM. Hemorrhagic encephalitis, mRS -2 (improved)

^5^17 years, male, Bethel: CASPR2 1:1000 in serum, CSF negative, VGKC complex 217 pM. Neuromyotonia, mRS -3 (improved)

^6^33 years, male, Bethel: CASPR2 1:500, CSF not studied, VGKC complex 332 pm. Myasthenia with thymus cancer, then neuromyotonia, mRS + 3 (deteriorated)

### Clinical ratings

Of 173 contacted institutions, 115 (66.5%) filled in the questionnaires for 413/576 patients (71.7%). The “yes/not sure/no” rating percentages for retrospectively assuming a CNS or PNS autoimmune disease were 58.4%/5.8%/35.3%. The proportions provided a grading of the antibody groups (Fig. 2d and Table 1).

#### NMDAR high

Positive clinical ratings and clinical data consistent with autoimmune encephalitis were provided in 42/44 cases. Two were rated by treating physicians as negative: a 65-year old man with no previous encephalitis had a 13-month history of mediotemporal lobe seizures of unexplained origin and mild verbal memory impairment. Serum titer was 1:2000. CSF contained 153 cells/µl and NMDAR antibodies at 1:12. No alternative cause was established but neither the syndrome nor sex or age of the patients fit to classical anti-NMDAR encephalitis. The patient was lost to follow-up. A 15-year old boy with multiple sclerosis (MS) harbored NMDAR antibodies in CSF at 1:12 (serum negative) < 1 month after the first disease signs; myelin oligodendrocyte glycoprotein (MOG) and aquaporin-4 (AQP4) antibodies were negative. He developed relapsing–remitting MS but never anti-NMDAR encephalitis.

#### NMDAR in serum but not in CSF

Seven patients were serum-only-positive in the CBA; TBA was negative with serum and with CSF. They had etiologically unclear focal epilepsy (54 years, female), progressive myoclonus epilepsy (not further specified, 3 years, male), focal epilepsy after presumed limbic encephalitis (72 years, female, viral encephalitis in her history), anaplastic astrocytoma III° (4 years, male), frontotemporal dementia with parkinsonism (71 years, male), presumed limbic encephalitis, status epilepticus and bone metastases (suspected bronchial carcinoma, 72 years, male), and dementia with depression (48 years, female).

#### NMDAR in serum and no CSF tested

Thirteen patients had only serum studied as their first material. Eight underwent subsequent CSF studies. CSF was NMDAR antibody positive in six, five of which were classified as anti-NMDAR encephalitis (one had a positive serum-TBA), whereas one had structural post-herpes epilepsy [39]; two were CSF negative (focal epilepsy, 35 years, male; progressive supranuclear palsy, 72 years, male). The remaining five had no CSF study (cerebellar syndrome, 57 years, female; glioblastoma, 50 years, female; polymorphic subjective complaints and non-epileptic twitches, 41 years, male; dementia with depression, 48 years, female; focal epilepsy, 57 years, male). No patient in this subgroup without anti-NMDAR encephalitis had a positive serum-TBA.

#### LGI1

All 81 patients had serum antibody titers ≥ 1:64; the 56 patients with available clinical diagnoses had limbic encephalitis with or without faciobrachial dystonic seizures (FBDS).

Five cases received the diagnosis of focal epilepsy of an unknown cause (in one case, with hippocampal sclerosis) at epilepsy centers. Physicians rated them as “negative” or “uncertain”. Serum titers were 1:128-1:6000. Disease duration was longer than in the cases with a positive clinical rating (2.3, 4.2, 12.0, 12.0, and 302.5 months versus median 1.6, IQR 0–162 months; *p* = 0.04, Mann–Whitney *U* test). They did not receive immunotherapy. Three patients with a follow-up remained unchanged. Apparently, the cognitive decline and the imaging features were not impressive. Therefore, treating physicians did not make the autoimmune encephalitis diagnosis. Finally, one 78-year old woman had severe encephalomyeloradiculitis and albumino-cellular dissociation in CSF, as in Guillain-Barré syndrome. She also developed dementia. Five months after disease onset, she had a serum titer of 1:1000. Despite steroids, she remained at mRS 5 (due to the neuropathy, no information on cognitive outcome).

#### CASPR2

Thirty-four out of 44 CASPR2 cases (77%) with available clinical data had frequent and well-known clinical associations: limbic encephalitis (*N* = 26), Morvan syndrome (*N* = 3), neuromyotonia (*N* = 4) or cerebellar ataxia (*N* = 1). Clinical ratings were available in 25 patients, and they were all considered “positive”.

Three cases were rated “uncertain” but had high serum titers (1:32,000–1:6,000,000) and the clinical diagnoses of chorea (66 years, male), encephalitis with brain stem plus cerebellar lesions and histopathologic evidence of encephalitis (64 years, male), and lesions of the optic nerves with loss of vision in the presence of a thymoma (46 years, female).

The remaining patients had other conditions and were not rated as having an autoimmune disease (negative, *N* = 8; uncertain, *N* = 1). Serum titers were 1:128–1:500. Diagnoses were chronic-inflammatory demyelinating polyneuropathy (76 years, male), focal epilepsy with hippocampal sclerosis (48 years, female), dementia with amyloid angiopathy (male, 83 years), delusional disorder (60 years, male), paranoid-hallucinatory psychosis (54 years, female), Creutzfeldt-Jakob disease (75 years, male), chronic progressive external ophthalmoplegia (65 years, female), Parry–Romberg syndrome (30 years, male), and hemorrhagic encephalitis (74 years, female).

#### GAD65 antibodies

Clinical syndromes were available from 111/119 cases: focal epilepsy, *N* = 58 (49%); limbic encephalitis, *N* = 23 (19%); progressive encephalomyelitis with rigidity and myoclonus (PERM)/stiff-man syndrome (SMS)/stiff-limb syndrome, *N* = 8 (7%); cerebellar ataxia, *N* = 7 (6%); epilepsy not further specified, *N* = 5 (4%); single seizure, *N* = 1 (1%); other, *N* = 9 (7%; 2 patients had dementia, both 81 years; encephalopathy, 50 years; MS, 17 years; psychosis, 48 years; ocular myasthenia gravis with antibodies against the muscular acetylcholine receptor and titin plus sarcoidosis of lung and skin, 38 years; unclear coma, 47 years; developmental retardation with macrocephalia and periventricular leukomalacia, 1 year; depressive syndrome, 37 years, the only male patient within the syndromic category “other”). There was almost equipoise in clinical ratings of “yes” (*N* = 52) versus “no”/”uncertain” (*N* = 41). Even within the frequent syndromes (limbic encephalitis, focal epilepsy, PERM/SMS, and cerebellar ataxia), positive and non-positive ratings were equal. Disease durations did not differ between patients with “positive” and “negative”/”uncertain ratings”. The dominance of females (93/119, 78%) was found across all syndromic groups.

#### Other antibodies, tumors and previous viral encephalitis

See Supplementary material.

### Immunotherapies and outcomes

**Fig. 3:**
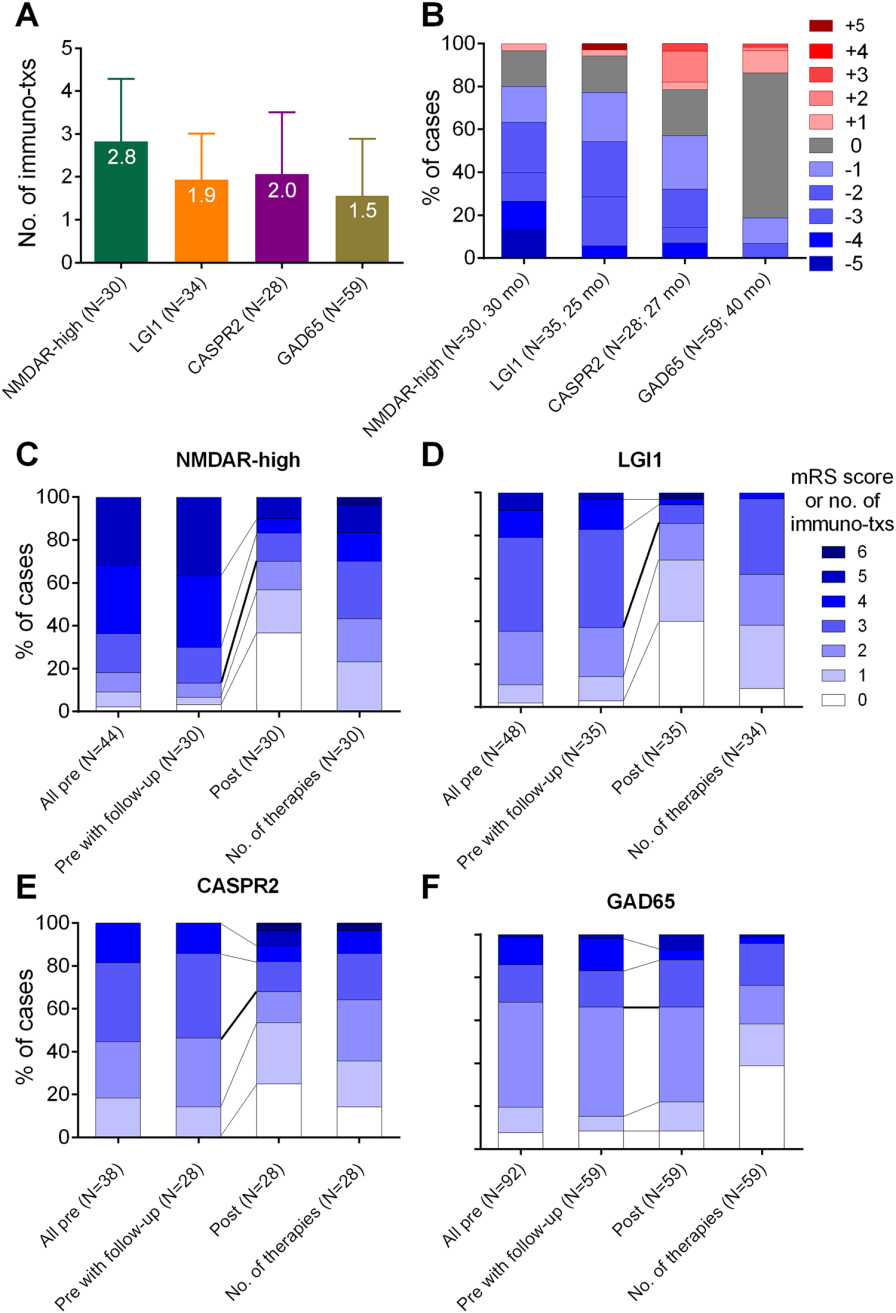
Immunotherapy and outcome in the four major antibody groups (NMDAR-high, LGI1, CASPR2, and GAD65). **a** Mean number of immunotherapies per patient with standard deviations. **b** Proportions and extent of changes according to the modified Rankin scale (mRS). Blue boxes indicate improvements, grey boxes stability, and reddish boxes deterioration. Labeling of the *x*-axis: after the *N* values, the median follow-up is given (mo = months). **c–f** Clinical performance (mRS) at antibody diagnostics (“pre”) and at most recent follow-up (“post”). Lower values indicate better performance. Left-most bars: all patients with rating at antibody diagnostics; second bars: patients with “post” ratings available at the time-point “pre” (there are no relevant differences between the total groups and those with an outcome); third bars: outcome; right-most bars: proportions of number of immunotherapies used. Please note that information on “no. of therapies” was only available for 34 LGI1 patients

A ≥ 3-month follow-up was available for 251 patients. These were rated after a mean of 32 months (SD 21, range 3–79 months) since antibody diagnosis and 23 months (SD 20, range 0–71 months) after last follow-up; 192 patients received at least one immunotherapy (Fig. 3a). Among all 244 patients with a ≥ 3-month follow-up and available outcome, 109 improved (44.7%). For patients with defined surface antibodies (AMPAR2, CASPR2, GABABR, GlyR, LGI1, or NMDAR-high), the ratio (72/102, 68.6%) was even better (Fig. 3b–f).

## Discussion

This large prospective series of > 10,000 patients was extensively and uniformly tested for neural antibodies in a routine laboratory with dedicated evaluators and a short turnover time. The general approach with an evaluation starting from the antibody findings in a routine diagnostic setting and not from patients with predefined features (this increases the likelihood of positive findings) [17, 22, 29] or selected antibodies [18, 26] is novel. Cases came from day-to-day practice and were considered clinically suspicious. However, selection criteria remained uncontrolled and were obviously not very stringent: only 5.3% of patients were antibody positive. The yield could be higher if patients were chosen in a more focused manner, e.g., according to Graus’ criteria of “possible autoimmune encephalitis” [20]. On the other hand, such an approach may miss cases with LGI1 antibodies and predominant focal seizures as reported above. CNS disorders prevailed (approximately two-thirds of samples). An overrepresentation of seizure disorders is possible; comparative figures from other laboratories are not available to our knowledge.

The four major antibody groups with defined targets were GAD65, LGI1, NMDAR-high, and CASPR2. Age, sex (Fig. 1), and IgG subclass distribution (Fig. 2c) were similar to previous publications [1, 23, 27, 41–43, 45]. Only half of the patients were tested within 6 months post-disease onset. The broad range of latencies suggests differential disease evolutions, fast for NMDAR, slow for GAD65 (Fig. 2a).

The Bethel VGKC complex, LGI1 and CASPR2 results had high interlaboratory reproducibility when verified in the Vienna laboratory. This suggests that the use of the Euroimmun assays in experienced hands provides reliable results, at least for these antibodies. Including VGKC complex antibody positives increased the a priori probability of finding LGI1 or CASPR2 antibody positives (more than just testing some of the many negatives). This gives the chance not only to replicate (or refute) positive findings but also to detect previously overlooked antibodies.

CSF was required for the diagnosis in 28% of NMDAR-high cases studied in serum–CSF pairs (Fig. 2b). This was previously reported (with 14% CSF-only NMDAR antibodies) [21].

Assessment of antibody validity is not easy, because there is no external gold standard. We did not exclude low-titer antibodies ante hoc (except in CASPR2 and GAD65 [6, 34]). Clinical ratings may be the best possible approximation. The retrospective judgement of the treating physicians (“autoimmune CNS or PNS disease”) provided the highest percentages of “positive” ratings for surface antibodies and onconeural antibodies (Fig. 2c). Groups with low percentages exhibited either equipoise in the interpretation (e.g., GAD65 antibodies: are they really indicative of an ongoing autoimmune process?), or physicians noted their conspicuous inhomogeneous nature (in VGKC complex, neuropil–CSF, and GlyR, specificity has been questioned in the literature [3, 40, 44]).

High-titer NMDAR antibodies (i.e., positive in CSF or a serum titer > 1:500) are very specific for anti-NMDAR encephalitis [21]. Patients ≤ 35 years and females are at a higher risk. In our series, 12% had ovarian teratomata. This is less than in North-American series. The probable reason is that patients of Afro-American and Asian descent are more frequent there. They more often have anti-NMDAR encephalitis with teratoma (> 40%) [42].

Serum-only reactivity with NMDAR-transfected cells (in the absence of neuropil staining in the TBA) at titers ≤ 1:500 is non-specific. This was previously inferred [20, 40], and it is formally demonstrated here. Only those patients who were subsequently found to have NMDAR antibodies in CSF, too, had typical anti-NMDAR encephalitis and responded to immunotherapy. Previous studies have at times considered low-titer serum-only findings as indicating specific autoimmune processes in patients with psychoses, other neurological and psychiatric diseases and potentially even healthy controls [8, 31]. Such interpretations have rightly been criticized for their lack of specificity [30]. This is supported by our findings.

LGI1 antibodies (all ≥ 1:64 in this study) were very specific: 50/56 patients had limbic encephalitis, in part with FBDS (89%). One elderly female had an autoimmune neuropathy but with dementia. Five patients with predominant recurrent focal seizures (in part for > 1 year) were seen at epilepsy centers and were neither diagnosed nor treated as autoimmune encephalitis. In contrast to most immunologically treated LGI1 patients, they did not improve. We speculate that these six patients had a forme fruste of limbic encephalitis; alternatively, they may have had a different disease, and the LGI1 antibodies may have been non-specific bystanders. People > 55 years had a higher likelihood of acquiring LGI1-antibody-associated diseases. Younger people, even in the pediatric age range, may occasionally be affected [37].

CASPR2 antibodies with a titer ≥ 1:128 are rather specific for limbic encephalitis, neuromyotonia, Morvan syndrome, or cerebellar ataxia. At times, CASPR2 antibodies occur with chorea, brainstem encephalitis plus cerebellitis, or less characteristic constellations like optic neuritis in the context of thymoma [18, 38]. CASPR2 antibodies are most often found in men > 55 years, as previously reported [23, 25, 28, 43]. Pediatric cases occur [37].

The majority of patients with GAD65 (> 80%) had focal epilepsy [32], limbic encephalitis [33], PERM/SMS [9], or cerebellar ataxia [2]. The almost equal positive versus negative/uncertain clinical rating ratios appear to be due to uncertainty how to interpret GAD65 antibodies. The preponderance of females suggests a genetic component. Antibody detection followed an inverted-U curve over the lifespan, with a peak at 40–45 years. This maximum was less marked than the peaks in the NMDAR-high, CASPR2, and LGI1 groups. The GAD65-CBA was recently found to produce reliable results in comparison to indirect immunohistochemistry or enzyme-linked immunosorbent assay [36].

Outcomes after ≥ 3 months were rated by the treating physicians (Fig. 3), an advantage over other studies, where it is not always clear who assessed the mRS. Among the four major groups, NMDAR-high and LGI1 patients had the largest proportions of improving cases and of mRS ≤ 2 (80% and 77%, respectively, and 70 and 83%; Fig. 2b). The rate of NMDAR-high patients with mRS ≤ 2 was similar to two large series (*N* = 252 after 2-year follow-up; *N* = 75 after a median follow-up of 27 months): 81% and 74%, respectively [13, 42]. In one study, the mRS ≤ 2 rate was 78% for LGI1 antibodies [13]. Of 28 CASPR2 patients, 57% improved after a median follow-up of 27 months. This is lower compared to two existing studies (94% of 16 patients after a median of 28 months [25] and 91% of 27 patients after a median of 36 months [43]). The reason for this difference is unknown. The 59 GAD65 patients had the poorest improvement rate of only 19%. To our knowledge, no figures for comparison are available from the literature.

Limitations are related to the study’s retrospective aspects. We did not get clinical information on all antibody-positive cases. The clinical assessment was not formalized. Our TBA was neither as sensitive as reported by in-house assays from other laboratories (many samples with clear-cut CBA results did not produce a neuropil staining) and it was also not very specific (the “neuropil staining” did not indicate many cases with autoimmune encephalitides). Control laboratory tests were only done for a subset of antibody-positive patients. Only a few negative materials were re-tested. We may have overlooked positive cases. The mRS may be insensitive to circumscribed cognitive problems, which makes the outcome of LGI1 patients look as good as NMDAR patients at follow-up; in fact, the LGI1 group has remaining memory problems that the NMDAR cases do not normally have.

In conclusion, test results for neural antibodies with commercially available assays in a dedicated routine laboratory are reliable (demonstrated for LGI1, CASPR2 and VGKC complex cases, if LGI1 or CASPR2 antibody positive or negative) and clinically meaningful. One should consider clinical presentation, demographic features, and titers when interpreting an antibody result in an individual patient. Patients positive for neural antibodies were in more than half of the cases retrospectively judged to have had an autoimmune condition. NMDAR-high, LGI1, GABABR, and AMPAR patients achieved positive ratings in ≥ 90% of cases. Of the patients with surface antibodies and follow-up, 64% improved. These data underscore the enormous therapeutic potential of this new branch of diagnostics.

## Electronic supplementary material

Below is the link to the electronic supplementary material.

Supplementary file1 (PDF 158 kb)
