# Correction to: Routine diagnostics for neural antibodies, clinical correlates, treatment and functional outcome

**DOI:** 10.1007/s00415-021-10634-2

**Published:** 2021-06-25

**Authors:** Christian G. Bien, Corinna I. Bien, Müjgan Dogan Onugoren, Desiree De Simoni, Verena Eigler, Carl-Albrecht Haensch, Martin Holtkamp, Fatme S. Ismail, Martin Kurthen, Nico Melzer, Kristina Mayer, Felix von Podewils, Helmut Rauschka, Andrea O. Rossetti, Wolf-Rüdiger Schäbitz, Olga Simova, Karsten Witt, Romana Höftberger, Theodor W. May

**Affiliations:** 1 Epilepsy Center Bethel, Krankenhaus Mara, Maraweg 17-21, 33617 Bielefeld, Germany; 2 Laboratory Krone, Bad Salzufen, Germany; 3 Department of Neurology, Epilepsy Center, Friedrich-Alexan Der-Universität, Erlangen-Nürnberg, Germany; 4 Division of Neuropathology and Neurochemistry, Department of Neurology, Medical University of Vienna, Vienna, Austria; 5 Department of Neurology, University Hospital St. Poelten, St. Poelten, Austria; 6 Department of Neurology, Städtisches Klinikum Ludwigshafen Am Rhein, Ludwigshafen, Germany; 7 Department of Neurology, Faculty of Health, Kliniken Maria Hilf Moenchengladbach, University of Witten/Herdecke, Moenchengladbach, Germany; 8 Epilepsy‑Center Berlin‑Brandenburg, Institute for Diagnostics of Epilepsy, Evangelisches Krankenhaus Königin Elisabeth Herzberge, Berlin, Germany; 9 Department of Neurology, University Hospital Bochum, Knappschaftskrankenhaus, Bochum, Germany; 10 Swiss Epilepsy Center, Zurich, Switzerland; 11 Department of Neurology With Institute of Translational Neurology, University Hospital Münster, Münster, Germany; 12 Department of Neurology, University Hospital of Augsburg, Augsburg, Germany; 13 Department of Neurology, University Medicine Greifswald, Greifswald, Germany; 14 Department of Neurology and Karl Landsteiner Institute for Neuroimmunological and Neurodegenerative Disorders, Sozialmedizinisches Zentrum Ost, Donauspital, Vienna, Austria; 15 Department of Clinical Neurosciences, University Hospital (CHUV) and University of Lausanne, Lausanne, Switzerland; 16 Department of Neurology, EvKB-Bethel, Bielefeld, Germany; 17 Protestant Hospital Alsterdorf, Epilepsy Center Hamburg, Hamburg, Germany; 18 Department of Neurology and Research Centre of Neurosensory Sciences, Carl Von Ossietzky University, Oldenburg, Germany; 19 Society of Epilepsy Research, Bielefeld, Germany

## Correction to: Journal of Neurology (2020) 267:2101–2114 10.1007/s00415-020-09814-3

The article Routine diagnostics for neural antibodies, clinical correlates, treatment and functional outcome, written by Christian G. Bien, Corinna I. Bien, Müjgan Dogan Onugoren, Desiree De Simoni, Verena Eigler, Carl‑Albrecht Haensch, Martin Holtkamp, Fatme S. Ismail, Martin Kurthen, Nico Melzer, Kristina Mayer, Felix von Podewils, Helmut Rauschka, Andrea O. Rossetti, Wolf‑Rudiger Schäbitz, Olga Simova, Karsten Witt, Romana Höftberger and Theodor W. May, was originally published Online First without Open Access. After publication in volume 267, issue 7, page 2101–2114 the author decided to opt for Open Choice and to make the article an Open Access publication. Therefore, the copyright of the article has been changed to © The Author(s) 2021 and this article is licensed under a Creative Commons Attribution 4.0 International License, which permits use, sharing, adaptation, distribution and reproduction in any medium or format, as long as you give appropriate credit to the original author(s) and the source, provide a link to the Creative Commons licence, and indicate if changes were made. The images or other third party material in this article are included in the article’s Creative Commons licence, unless indicated otherwise in a credit line to the material. If material is not included in the article’s Creative Commons licence and your intended use is not permitted by statutory regulation or exceeds the permitted use, you will need to obtain permission directly from the copyright holder. To view a copy of this licence, visit http://creativecommons.org/licenses/by/4.0/.

Open Access funding enabled and organized by Projekt DEAL.

The original article has been corrected.

