# Supplementary material for: Routine diagnostics for neural antibodies, clinical correlates, treatment and functional outcome
Source: J Neurol. 2020 Apr 3;267(7):2101–14. doi: 10.1007/s00415-020-09814-3 (PMC8213550; doi:10.1007/s00415-020-09814-3)
Supplement: Supplementary file 1 — Supplementary file1 (PDF 158 kb) [file 415_2020_9814_MOESM1_ESM.pdf]

Rarer antibodies: data and discussion

Onconeural antibodies without a tumor

Onconeural antibodies were detected by a positive band on an immunoblot, following the manufacturer's instructions (Ravo, Freiburg, Germany), with the following antigens: Hu, Yo, Ri, CV2, amphiphysin, Ma2, and Sox1. Antibody reactivity was only noted as the diagnosis if a corresponding staining pattern was seen in mouse brain [1].

Among eight patients, six received a negative clinical rating even though they had typical paraneoplastic syndromes (Yo, cerebellar disease, female, N=2; Ma2, limbic encephalitis, N=2; Hu with Sox1 and Hu with CV2, both encephalitis with seizures); five of them had disease durations ≤8 months. Perhaps physicians did not subsume paraneoplastic conditions under "autoimmune" diseases. The other two patients with negative ratings had uncommon syndromes: CV2, 69 years, female, motor neuron disease, antibodies detected at 8 months, no follow-up; amphiphysin, 38 years, male, focal epilepsy, antibodies detected after 2.6 years, total disease duration at most recent follow-up 18 years, still no tumor or disease evolution.

Onconeural antibodies predict a paraneoplastic condition. The 6 cases without known neoplasm but typical syndromes are consistent with the observation that tumors may be detected only years after the antibodies [1]. The significance of the cases with CV2 or amphiphysin antibodies in patients without a tumor and atypical syndromes remains unexplored.

γ-aminobutyric acid-B receptor (GABABR)

Syndromes and clinical ratings were available from 12 patients: limbic encephalitis (N=9), cerebellar syndrome (N=1), myelitis (N=1), Lambert-Eaton myasthenic syndrome (N=1). Eleven were rated positive, and eight were paraneoplastic. Two patients improved, five remained stable, two deteriorated (died), and in three, no outcome was available. Serum titers were 1:32-1:50000. Even the five patients with low titers (≤1:64) were rated as clinically positive.

The GABABR group behaved largely as described in previous series [2-4]. Low titers do not apparently contradict specificity.

Glycine receptor (GlyR)

Among the ten GlyR patients, two had progressive encephalomyelitis with rigidity and myoclonus (PERM; 65 years, male, serum 1:128, CSF 1:4) or Stiff-man syndrome (SMS; 75 years, male, serum 1:250, CSF not available [n.a.]). The others had autoimmune encephalitis

that was not further specified (N=2: 54 years, male, serum negative, CSF 1:4; 58 years, female, serum 1:128, CSF negative), epilepsy (N=3: 4 years, male, serum 1:500, CSF n.a..; 24 years, female, serum 1:32, CSF n.a.; 72 years, female, serum 1:500, CSF negative, with a tumor), polyneuritis with antibodies against GQ1b and muscle-specific kinase (47 years, male, serum 1:16, CSF n.a.), vasovagal syncope (74 years, female, serum 1:16, CSF n.a.); psychogenic non-epileptic seizures (30 years, female, serum 1:1000, CSF n.a.).

GlyR patients had a wide range of clinical manifestations. Apart from PERM/SMS, which seem robustly correlated with GlyR antibodies, these features may indicate immune activation but are otherwise non-specific [5]. Titer level did not help discriminate specific from less-specific occurrences.

Voltage-gated potassium channel (VGKC) complex, not LGI1 or CASPR2

VGKC complex antibodies were determined in serum with a radioimmunoprecipitation assay (RIA) at the Laboratory Krone (Bad Salzuflen, Germany) according to the manufacturer's instructions (RSR Ltd, Cardiff, UK). Concentrations ≥100 pmol/l were considered elevated [6]. Not all senders asked for VGKC complex antibody testing.

In this large group (N=90), there were 21 positive, 6 uncertain, and 39 negative ratings (24 cases [27%] not rated). VGKC complex antibody concentrations and disease durations (median 6, mean 58, range 1-402 months) did not differ among these subgroups. Clinical syndromes, however, differed: patients with disorders presumably of limbic origin (often epileptic) or cerebellar disorders were most prominent in the positive group (18/21, 86%), intermediate in the uncertain group (4/6, 67%), and rare in the negative group (8/39, 21%).

In the large VGKC complex group (N=90), physicians rated approximately one-third of the cases as autoimmune despite the recent data speaking against specificity [7] and suggesting binding to intracellular epitopes [8]. Their ratings appear to reflect the previous assumption of a specific association with limbic encephalitis [6] or cerebellar ataxia [9]. The broad range of associated conditions in this series underlines that these antibodies are non-specific. We removed the VGKC-RIA from our standard diagnostic battery.

Neuropil antibodies in CSF but no specific antibody found on the cell-based assays

There were no specific, dominant, or characteristic findings regarding disease duration, clinical features, or outcome.

Neuropil antibodies in CSF were associated with very divergent clinical presentations. Our diagnostic criteria at the microscope were probably too liberal and led to inclusion of cases with "sticky" CSF samples. We are now more stringent and accept only clear-cut neuropil patterns in CSF. Surface binding to live neurons, a technique available in research laboratories, may help to increase specificity [10].

Table 1: Patients with a tumor

Antigens	Patients with tumor	Patients with data available	%
Onconeural	18	26	69%
GABABR	8	12	67%
Neuropil-CSF	9	46	20%
VGKC complex	11	63	17%
NMDAR-high	7[1]	43	16%
CASPR2	5	38	13%
NMDAR in serum only	2[2]	17	12%
GlyR	1	10	10%
GAD	5	89	6%
LGI1	2	47	4%

Table 2: Patients with previous viral encephalitis

Antigens	Patients with previous encephalitis	Patients with data available	%
NMDAR-high	7	40	18%
NMDAR in serum only	2	18	11%
Neuropil-CSF	5	48	10%
VGKC complex	3	61	5%
GAD	3	83	4%

[1]5 teratomata

[2] no teratoma

References

1. Graus F, Delattre JY, Antoine JC, *et al.* Recommended diagnostic criteria for paraneoplastic neurological syndromes. *J Neurol Neurosurg Psychiatry*. 2004 **75:** 1135-1140.
2. Lancaster E, Lai M, Peng X, *et al.* Antibodies to the GABA(B) receptor in limbic encephalitis with seizures: case series and characterisation of the antigen. *Lancet Neurol*. 2010 **9:** 67-76.
3. Höftberger R, Titulaer MJ, Sabater L, *et al.* Encephalitis and $GABA_B$ receptor antibodies: Novel findings in a new case series of 20 patients. *Neurology*. 2013 **81:** 1500-1506.
4. Jeffery OJ, Lennon VA, Pittock SJ, Gregory JK, Britton JW, McKeon A. GABAB receptor autoantibody frequency in service serologic evaluation. *Neurology*. 2013 **81:** 882-887.
5. Armangue T, Sabater L, Torres-Vega E, *et al.* Clinical and Immunological Features of Opsoclonus-Myoclonus Syndrome in the Era of Neuronal Cell Surface Antibodies. *JAMA Neurol*. 2016 **73:** 417-424.
6. Vincent A, Buckley C, Schott JM, *et al.* Potassium channel antibody-associated encephalopathy: a potentially immunotherapy-responsive form of limbic encephalitis. *Brain*. 2004 **127:** 701-712.
7. van Sonderen A, Schreurs MW, de Bruijn MA, *et al.* The relevance of VGKC positivity in the absence of LGI1 and Caspr2 antibodies. *Neurology*. 2016 **86:** 1692-1699.
8. Lang B, Makuch M, Moloney T, *et al.* Intracellular and non-neuronal targets of voltage-gated potassium channel complex antibodies. *J Neurol Neurosurg Psychiatry*. 2017 **88:** 353-361.
9. Becker EB, Zuliani L, Pettingill R, *et al.* Contactin-associated protein-2 antibodies in non-paraneoplastic cerebellar ataxia. *J Neurol Neurosurg Psychiatry*. 2012 **83:** 437-440.
10. Rosenfeld MR, Titulaer MJ, Dalmau J. Paraneoplastic syndromes and autoimmune encephalitis: Five new things. *Neurol Clin Pract*. 2012 **2:** 215-223.
